# Signalling and regulation of plant development by carbon/nitrogen balance

**DOI:** 10.1111/ppl.70228

**Published:** 2025-04-23

**Authors:** Iris Fañanás‐Pueyo, Gerardo Carrera‐Castaño, Mónica Pernas, Luis Oñate‐Sánchez

**Affiliations:** ^1^ Centro de Biotecnología y Genómica de Plantas, Universidad Politécnica de Madrid (UPM) ‐ Instituto Nacional de Investigación y Tecnología Agraria y Alimentaria (INIA/CSIC), Campus de Montegancedo UPM Pozuelo de Alarcón (Madrid) Spain; ^2^ Departamento de Biotecnología‐Biología Vegetal Escuela Técnica Superior de Ingeniería Agronómica, Alimentaría y de Biosistemas, UPM Madrid Spain

## Abstract

The two most abundant macronutrients in plant cells are carbon (C) and nitrogen (N). Coordination of their cellular metabolism is a fundamental factor in guaranteeing the optimal growth and development of plants. N availability and assimilation profoundly affect plant gene expression and modulate root and stem architecture, thus affecting whole plant growth and crop yield. N status also affects C fixation, as it is an important component of the photosynthetic machinery in leaves. Reciprocally, increasing C supply promotes N uptake and assimilation. There is extensive knowledge of the different mechanisms that plants use for sensing and signalling their nutritional status to regulate the assimilation, metabolism and transport of C and N. However, the crosstalk between C and N pathways has received much less attention. Plant growth and development are greatly affected by suboptimal C/N balance, which can arise from nutrient deficiencies or/and environmental cues. Mechanisms that integrate and respond to changes in this specific nutritional balance have started to arise. This review will examine the specific responses to C/N imbalance in plants by focusing on the main inorganic and organic metabolites involved, how they are sensed and transported, and the interconnection between the early signalling components and hormonal networks that underlies plants’ adaptive responses.

## 1 INTRODUCTION

Plant growth and yield rely on nutrient‐signalling pathways efficiently coordinated with development and environmental conditions. The two most abundant macronutrients in plant cells are carbon (C) and nitrogen (N), and responses to either of these nutrients have been studied extensively. However, the balance between C‐ and N‐compounds has received less attention despite the fact that the C/N ratio, in addition to the C status or the N availability alone, significantly affects plant growth and development (Martin et al., 2002). The control of C and N interaction is assisted by a complex network of signals emanating from nitrate (NO_3_^‐^), ammonium (NH_4_^+^), and N‐containing metabolites as well as from C metabolism (Coruzzi & Zhou, 2001; Miller et al., 2008; Stitt & Krapp, 1999). Subsequently, sensing and transport of these signals are coordinated with several gene regulatory networks to maintain global C/N homeostasis in the plant.

**FIGURE 1:**
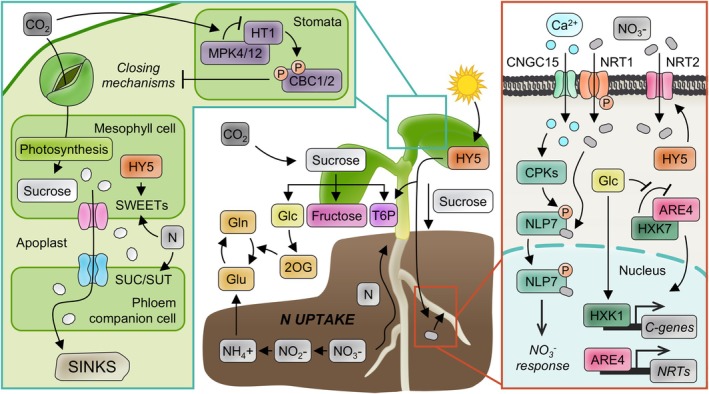
Schematic representation of sensors, transporters and major signalling players that underlie C/N balance regulation. Central panel depicts the coordination between C fixation and N assimilation. Left panel illustrates CO_2_ sensing, fixation and control of stomatal opening, as well as the downstream transport of sucrose from leaves to sink organs, influenced by N levels. Right panel shows NO_3_ uptake, transport and cytoplasmic and nuclear signalling in the root cells in response to N availability. Finally, the mechanism that coordinates glucose (Glc) signalling and N utilization in rice, involving HKX7‐ARE4 TF complex and translocation to the nucleus, is also depicted.

C is assimilated through photosynthesis from CO_2_ and the resulting sugars, in particular sucrose and glucose, are converted through glycolysis and the tricarboxylic acid cycle (TCA) to provide energy and C‐skeletons, such as 2‐oxoglutarate (2‐OG; also known as α‐ketoglutarate), for amino acid biosynthesis. N nutrients include inorganic (NO_3_^‐^ and NH_4_^+^) and organic compounds (i.e., amino acids), the latter being synthesized by incorporating NH_4_^+^ into the C‐skeletons. NO_3_^‐^ is first reduced by the nitrate reductase to nitrite (NO_2_^‐^) and then by nitrite reductase to NH_4_^+^. Next, NH_4_^+^ is incorporated into glutamate (Glu) to generate glutamine (Gln). Gln is converted back to Glu using 2‐oxoglutarate (2‐OG), an intermediate of the tricarboxylic acid cycle (TCA). Glu serves as a N‐donor for the biosynthesis of amino acids and other N‐containing compounds (Figure 1).

N assimilation is highly energy demanding and, in leaves, the photosynthetic metabolism is responsible for providing reducing power and ATP, whereas it is the oxidative pentose phosphate pathway that provides NADPH for biosynthesis in roots. Moreover, large amounts of N are invested in the photosynthetic machinery, as it requires enzymes built of amino acids. In addition, in land plants, the sites where C and N are absorbed are spatially separated. NO_3_^‐^ and NH_4_^+^ are taken up by the root system and assimilated both in roots and leaves, while C assimilation occurs in the leaf. Therefore, keeping appropriate C/N ratios throughout the plant requires a spatial exchange of energy and C‐skeletons with N‐compounds supported by dedicated sensing, signalling and transport mechanisms. This intricate relationship explains the interrelated effects produced by changes in C/N ratios. For example, N‐deficiency negatively affects the photosynthetic output, an effect reverted by increasing N levels. Concomitantly, illuminated and photosynthetically active leaves support higher levels of N assimilation compared to dark conditions (Matt et al., 2001; Nunes‐Nesi et al., 2010; Reed et al., 1983; Zheng et al., 2009).

Here, we focus on the different aspects of C and N interactions and their connections to reveal the complexity of the mechanisms underlying the regulation of their balance in plants. Thereby, we have reviewed major findings on the role of key C‐ and N‐compounds, their sensors and transporters, and their early signalling events to highlight their relationships and impact on the regulation of the C/N balance in plants. To facilitate reading, we have mostly used gene acronyms throughout the text and a list of complete gene names and their abbreviations have been included separately (Table S1).

## 2 SUGAR AND NITROGEN SIGNALS

The control of C and N interaction involves signals emanating from C metabolism and inorganic and organic N‐compounds. Although a certain number of molecules signalling C and N are known, we will focus in this section on the direct and essential signalling molecules involved in the interaction of both macronutrients.

Concerning C, atmospheric CO_2_ is fixed in mature leaves to generate sugars (Smith & Stitt, 2007). Plants are able to sense changes in the CO_2_ concentration in the intercellular air spaces of leaves throughout the day and respond by opening and closing their stomata (Zhang et al., 2018). Many studies have investigated the effect of elevated CO_2_ on plant nutrient uptake, especially on N. The results of these studies are highly variable, but most of them show that high CO_2_ concentration significantly reduces the efficiency of N acquisition by decreasing the rate of N uptake (Gojon et al., 2023; Soussana et al., 2005; Taub & Wang, 2008). However, the effect of high CO_2_ on N uptake seems to be different depending on the N source, affecting more negatively NO_3_^‐^ uptake than NH_4_^+^ (Rubio‐Asensio & Bloom, 2017). This effect has been described in several species like *Arabidopsis thaliana*, wheat (Bloom et al., 2010), *Medicago truncatula* (Guo et al., 2013), or rice (Shimono & Bunce, 2009). Regarding Arabidopsis, Li et al. (2008) reported that elevated CO_2_ enhanced the expression of transcripts of glycolytic and TCA cycle enzymes, downregulated plastid functions and modulated the expression of N metabolism genes, which resembled N‐deficiency conditions. Moreover, elevated CO_2_ promoted the storage and consumption of C‐compounds, including several organic acids of the TCA cycle. However, the increase in organic acids, substrates for transamination, did not result in increased amino acid accumulation except for aromatic amino acids. Also, high CO_2_ produced a dramatic increase of heat shock proteins and transcripts of glutathione transferases (Li et al., 2008), resembling an abiotic stress response. Authors argued that increased photosynthetic CO_2_ fixation alters the apparent C/N balance and this imbalance seems to constrain the possible growth benefits from elevated CO_2_. These effects are more pronounced under N deficiency, producing a decreased shoot/root ratio, reduced NO_3_^‐^ content and accumulation of anthocyanin in seedlings (Takatani et al., 2014). Additionally, in mature plants, high CO_2_ under N deficiency also promotes leaf chlorosis, anthocyanin accumulation and increased senescence‐associated gene expression (Aoyama et al., 2014). Indirect and physiological processes may explain the negative effects of elevated CO_2_ on N uptake and assimilation. For example, high CO_2_ triggers the closure of the stomata, which produces a reduction in stomatal conductance and transpiration. This could result in lower translocation of nutrients via the xylem and reduced acquisition of nutrients via roots (Gojon et al., 2023; Tanner & Beevers, 2001). This could also be explained as a consequence of an excessive accumulation of C‐compounds (Li et al., 2008; Krämer et al., 2022). Nevertheless, a CO_2_‐sensor has recently been identified, thus emerging as a possible direct signalling pathway (Takahashi et al., 2022; see sensors and transporters section).

Plant trioses phosphate synthesized by photosynthetic activity are converted into transient starch in chloroplasts or converted to sucrose in the cytoplasm through a series of enzymatic reactions (McClain & Sharkey, 2019). Sucrose is then transported to sink organs and stored in vacuoles or cleaved into fructose and glucose or UDP glucose. Out of these compounds, glucose and sucrose are the main sugar signals and they can show hormone‐like activities, being directly sensed and producing some direct effects on N signalling or metabolism (Li et al., 2021a; Sakr et al., 2018). For example, in Arabidopsis, the expression of key genes of NO_3_^‐^ uptake, reduction and assimilation is enhanced by exogenously supplied sugar, which is partly dependent on the glucose sensor HEXOKINASE1 (HXK1)‐mediated signalling (Lejay et al., 2003; Price et al., 2004; Figure 1). In rice, upon glucose perception, a mechanism involving the glucose sensor HXK7 and the ARE4 transcription factor (TF) activates the expression of a subset of high‐affinity NO_3_^‐^ transporter genes (Ma et al., 2023a; see sensors and transporters; Figure 1). On the other hand, excessive accumulation of glucose in response to high C availability leads to repression of photosynthetic genes and produces stomatal closure in an HXK1‐dependent manner, which entails reduced nutrient acquisition (Kelly et al., 2012, 2013). All these mechanisms seem to contribute to the maintenance of C/N balance in the plant. Interestingly, the impact of high C availability on plant development seems to be dependent on N levels. It was observed that a high concentration of glucose (6%) on MS media (N sufficiency) produces seedling growth arrest involving abscisic acid (ABA) and ethylene signalling. However, in the absence of N, a lower concentration of 2% glucose resulted in a similar developmental arrest without involvement of ABA or ethylene signalling (Cho et al., 2010; Lu et al., 2015a; Pourtau et al., 2004). This situation (low N and 2% glucose supply) induced leaf yellowing and changes in gene expression with high similarity to those observed during developmental senescence (Pourtau et al., 2004, 2006; Wingler et al., 2004, 2009). During leaf senescence, N from photosynthetic proteins of old leaves is exported and reused for the photosynthetic apparatus of younger organs or stored in seed storage proteins. Although photosynthesis declines during this process, in the absence of strong sinks, old leaves can accumulate sugars and signal an excess of C relative to low N availability (Wingler, 2018), suggesting that this situation might provoke a high C/N stress response.

The other major plant monosaccharide, fructose, has also been identified as a specific sugar signal. It was first noticed from observations that psicose, a fructose analogue, inhibits lettuce root growth in an HXK1‐independent manner (Kato‐Noguchi et al., 2005). Thus, specific components of fructose signalling, independent of HXK1‐mediated glucose signalling, have been identified. These components included the NAC089 TF (Li et al., 2011) and the cytosolic FINS1 protein (fructose‐1,6‐bisphosphatase; see sensors and transporters section). Like glucose, the addition of exogenous fructose to the medium produces seedling growth arrest in Arabidopsis (Cho & Yoo, 2011). But interestingly, and unlike glucose, decreasing the concentration of fructose in the absence of N did not trigger developmental arrest and had little effect on Arabidopsis seedling growth, suggesting that fructose can signal independently of glucose and N (Cho & Yoo, 2011). Additionally, it seems that fructose might also be a shoot‐to‐root signal for the start of the light period since it peaks in the early morning and is low during the rest of the diurnal cycle (Brauner et al., 2018).

Sucrose, like glucose, also produces seedling growth arrest depending on N availability (Gao et al., 2008). However, there are several sucrose‐specific responses unique to plants and unrelated to those produced by its derivates or by HXK1 (Chiou & Bush, 1998; Loreti et al., 2000; Wind et al., 2010). For example, sucrose downregulation of *Beta vulgaris* sucrose transporter *SUT1* is not elicited by hexoses and is not impeded by an HXK inhibitor (Chiou & Bush, 1998; Vaughn et al., 2002). Also, sucrose‐mediated *PAP1* induction, a TF involved in anthocyanin metabolism, is not produced by other sugars (Teng et al., 2005). In this case, the mechanism of sucrose perception remains unknown, although several candidates have been proposed. For instance, there is a pseudo‐perception mechanism dependent of sucrose (and not hexose) concentration based on nascent peptide‐mediated ribosome stalling at the stop codon of the Arabidopsis uORF2 upstream of the bZIP11 TF (Rahmani et al., 2009). This mechanism has a direct connection with N signalling since bZIP11 reprograms amino acid metabolism in response to sucrose (Hanson et al., 2008). A novel sucrose signalling pathway has been proposed linking plant response to constant changes in sucrose status with photosynthesis, and consequently control of plant growth and development (Nguyen et al., 2016).

Like sucrose, trehalose 6‐phosphate (T6P) is a non‐reducing disaccharide. During the evolution of higher plants, sucrose became the most common sugar and T6P (intermediate in the synthesis of trehalose) evolved a new role as ‘plant insulin’ by signalling how much sucrose is available for growth. T6P levels in plants increase along with sucrose levels and the first regulates the production of the latter (Figure 1). If the plant is producing too much sucrose during photosynthesis, T6P signals to invest some fixed C into organic acids and amino acids. At night, T6P also functions as an inhibitor of starch breakdown to control the kinetics of sucrose synthesis and avoid the complete depletion of starch reserves (Baena‐González & Lunn 2020; Fichtner et al. 2021a; Figueroa & Lunn 2016). Regarding T6P interaction with N, there are three direct connexions. First, in high sugar/low N seedling growth arrest experiments, endogenous T6P concentration, and not glucose, correlates with C and N availability in the medium (Li et al., 2020b). Second, high T6P levels stimulate NO_3_^‐^ assimilation and synthesis of organic acids to generate C skeletons for amino acid synthesis (Figueroa et al., 2016). Third, flowering in short‐day conditions relies on T6P signalling under low N (Olas et al., 2019).

In addition, to serve as C skeletons for amino acid synthesis, the organic acids generated in the TCA cycle are also precursors of hormones and other signalling molecules in plants. A recent work has suggested that these organic acids from the TCA cycle can act as hormone‐like molecules and activate signalling pathways. Thus, exogenous addition of some of them (i.e., succinate and α‐ketoglutarate) produces distinct root phenotypes in stem cell divisions and growth, and these phenotypes are not sufficiently explained by changes in ATP or ROS levels (Zhang et al., 2023). In any case, these effects and a possible connection with N or nutritional status remain unexplored.

Finally, NO_3_^‐^ is a positive signal for the induction of sugar unloading and assimilation, and several key genes for C metabolism, like pyruvate kinase, phosphoenolpyruvate carboxylase, 6‐phosphogluconate dehydrogenase, and glucose‐6‐phosphate dehydrogenase, are induced by NO_3_^‐^ (Scheible et al., 2004; Wang et al., 2003). NO_3_^‐^, as well as NH_4_^+^ and amino acids, activate the TOR kinase (Cao et al., 2019; Liu et al., 2021; see C/N signalling section), but the direct molecular links between different N sensors and N‐signalling pathways responsible for TOR activation are still unknown in plants (Liu et al., 2021).

## 3 SUGAR AND NITROGEN SENSORS AND TRANSPORTERS

Maintaining an appropriate balance or ratio of C and N involves the sensing and signalling of C and N status throughout the plant organs. NO_3_^‐^ and/or NH_4_^+^ are usually taken up by the root system and NO_3_^‐^ is transported to the leaf. Meanwhile, C is assimilated in the form of sucrose, which is the main long‐distance transported sugar, and exported from the source leaves to sink organs via phloem (Figure 1). Assimilated N is converted to amino acids, transiently stored in source leaves and roots and relocated to sink organs (Lalonde et al., 2004; Sakr et al., 2018). After assimilation, N is mainly transported to the mesophyll cells through the xylem in the form of Gln, Asp, Glu, and Asn for C assimilation. Thus, sink organs, such as flowers, fruits, seeds, and younger leaves, are the primary reserve for C and N assimilates, while source organs, such as older leaves and green plant parts, export carbohydrates produced by photosynthesis. The CO_2_ assimilation capacity is mainly affected by the N content of the leaves and is generally positively correlated with it. Under N deficiency conditions, leave's chlorophyll content and photosynthetic rate decrease. In consequence, plants have developed sensing mechanisms to control their photosynthetic output in response to the levels of C and the status of N in the soil and in their root system (Wingler et al., 2018; Xuan et al., 2017). The mechanisms underlying sugar and NO_3_^‐^ sensing are complex and involve different N and sugar transporters. These proteins must coordinate the N assimilation with C metabolism, nutrient availability and other environmental factors (Zheng et al., 2009).

At the front line, plants are able to sense changes in the CO_2_ concentration, and elevation of leaf CO_2_ concentration triggers a rapid stomatal closing, leading to reduced transpiration. Changes in transpiration have been associated with alteration in translocation of nutrients and their acquisition through the roots (Gojon et al., 2023). Sensing of CO_2_ involves negative regulators of high CO_2_‐induced stomatal closure, the HT1 (Hashimoto et al., 2006), CBC1 and CBC2 kinases (Hiyama et al., 2017), and positive regulators of early CO_2_ signal transduction, the MPK4/MPK12 kinases (Tõldsepp et al., 2018). Very recently, it was discovered that MPK4/12 and HT1 constitute the long‐sought primary stomatal CO_2_ sensor upstream of the CBC1 kinase. Thus, increased CO_2_ levels trigger the interaction between MPK4/MPK12 and HT1, which inhibits HT1 kinase activity and promotes stomatal opening. At low CO_2_, HT1 phosphorylates and activates the downstream CBC1 kinase (Takahashi et al., 2022; Figure 1).

Plants have evolved a complex mechanism to sense different sugars, including sucrose, glucose and fructose, causing a response that is sometimes specific to the type of sugar (Rolland et al., 2006; Smeekens & Hellmann, 2014). Sucrose is the main form of C; it is synthesized in the cytoplasm of mesophyll cells and initially transported out of mesophyll cells into apoplast via SWEET transporters (Chen et al., 2012). Then, sucrose is imported into companion cells from the apoplast through membrane‐localized SUTs/SUCs symporters (Scofield et al., 2007; Figure 1). While SUT/SUC family transporters studied to date, including vacuolar SUTs, are sucrose/H+ symporters, SWEETs are able to independently transport sucrose and fructose across cell membranes (Gautam et al., 2022; Hu et al., 2021; Jeena et al., 2019). SWEETs also participate in abiotic stress responses by maintaining intracellular sugar concentrations (Lu et al., 2019; Mathan et al., 2021; Miao et al., 2017). Interestingly, some SWEET members have also been demonstrated to transport hormones in addition to sugars. Specifically, two Arabidopsis *SWEET* genes (*AtSWEET13* and *AtSWEET14*) are involved in the transportation of various forms of gibberellins (GAs; Kanno et al., 2016). Sucrose transport and N levels need to be coordinated to maintain C/N homeostasis. It has been described that the transport from source to sink mediated by different sugar transporters is more efficient at sufficient than at deficient N levels. Accordingly, N levels affect the expression of *SWEETs* and *SUTs* in different plant species like Arabidopsis, maize or rice. *For instance, AtSWEET16 expression decreases under low N levels and its overexpression enhances growth and* N content at adequate N supply but not under N deficiency (Klemens et al., 2013). Interestingly, it has been showed that enhanced expression of *OsSWEET11* and *OsSWEET14* increased grain filling and grain yield even under low N conditions in rice (Fei et al., 2021). In barley, *HvSWEET11* expression is induced more efficiently by NH_4_^+^ than by NO_3_^‐^, suggesting a differential role of SWEET transporters depending on the source of N (Lopes et al., 2008). Finally, *AtSWEET17* modulates fructose balance under different abiotic stresses, including low N (Chardon et al., 2013; Guo et al., 2014). In summary, N status seems to regulate the functionality of some of the sugar transporters to balance C/N levels by affecting their gene expression and protein levels.

The evolutionary conserved HXK1 hexokinase family participates in sugar metabolism and sensing (Moore et al., 2003). This family of proteins catalyse the phosphorylation of hexose sugars in the cytoplasm as the first step of the glycolytic pathway. However, Arabidopsis HXK1 can function as a glucose sensor, independently of its catalytic activity, by forming a nuclear complex with the vacuolar H+‐ATPase B1 (VHA‐B1), the 26S proteasome AAA‐ATPase subunit (RPT5B), and putative TFs (Cho et al., 2006). Thus, HXK1 interacts with these proteins in the nucleus to control transcription and diverse glucose responses. Under high glucose levels, the HXK‐mediated pathway inhibits photosynthesis and transpiration as well as seedling development but, under N sufficiency, HXK1 induces growth in vegetative and reproductive organs (Cho et al., 2007; Vanderwall et al., 2023). Recently, in rice, the glucose sensor OsHXK7 has been found to form a complex with the ARE4 TF in the cytoplasm. Upon sensing a glucose signal, ARE4 is released and translocated into the nucleus to activate the expression of a subset of high‐affinity NO_3_^‐^ transporter genes to increase NO_3_^‐^ uptake and accumulation (Ma et al., 2023b; Figure 1). Thus, this mechanism coordinates glucose signalling and N utilization in rice. Interestingly, this mechanism displays a diurnal pattern in response to circadian changes of soluble sugars (Ma et al., 2023a).

Fructose is also one of the prevalent sugars in plants (Kato‐Noguchi et al., 2005). Fructose signalling induces seedling developmental arrest and interacts with plant stress hormone signalling in a similar manner than glucose. Under N deficiency, fructose overaccumulates in leaves (Chardon et al., 2013) and exerts its regulation on seedling growth through FINS1, a cytosolic fructose 1,6‐bisphosphatase, whose function in fructose signalling is independent of its catalytic activity in sugar metabolism (Cho & Yoo, 2011). FINS1 protein has been suggested to be a fructose sensor capable of locating to the nucleus, where it interacts with WUS/WOX TFs to bind *WUS/WOX* promoters and regulates their activity to control stem cell function in a fructose dose‐dependent manner (Cho & Yoo 2011; Li et al., 2023).

NO_3_^‐^ is the main form of N absorbed by plants and, similarly to some sugars, it acts as a signal in plant physiology and development (Wang et al., 2018b). NO_3_^‐^ is absorbed from the soil into root cells, usually by the dual‐affinity NRT1 and high‐affinity NRT2 transporters (Chopin et al., 2007; Liu et al., 2022b; O'Brien et al., 2016; Tsay et al., 1993). NRT1.1/NPF6.3 (hereafter NRT1.1) is one of the most well‐studied members of its family and was initially characterized as a transporter involved in NO_3_^‐^ uptake by roots as well as root‐to‐shoot NO_3_^‐^ translocation (Léran et al., 2013; Liu et al., 1999). Its affinity for NO_3_^‐^ can change in response to substrate availability. Thus, phosphorylation of a threonine residue, Thr101, switches NRT1.1 from low‐ to high‐affinity state (Liu & Tsay, 2003). Later, it was shown that NRT1.1 functions as a NO_3_^‐^ sensor independently of its function as a transporter (Ho et al., 2009). It is involved in NO_3_^‐^‐dependent gene expression and root development and its ability to sense NO_3_^‐^ concentrations depends on its phosphorylation state. Thus, the CIPK23 kinase phosphorylates NRT1.1 to maintain a low‐level primary response under low NO_3_^‐^ concentration (Ho et al., 2009). On the contrary, ABI2 phosphatase can dephosphorylate CIPK23, thereby suppressing its kinase activity, thus enhancing NRT1.1 activity (Léran et al., 2015). Subsequently, the association of the NRT1.1 with the cyclic nucleotide‐gated channel protein (CNGC15) promotes the Ca^+2^ influx through the cell membrane (Wang et al., 2021c). Then, several Ca^+2^‐dependent kinases (CPK10/30/32) are able to induce the phosphorylation of the NLP7 TF to promote its shuttling from cytoplasm to the nucleus (Liu et al., 2017). NLP7 activates primary NO_3_^‐^ response genes (Konishi et al., 2013; Liu et al., 2022a) and it has been shown to be the primary NO_3_^‐^ sensor in plants. Upon binding to NO_3_^‐^, NLP7 suffers a conformational change favoring its nuclear retention and NO_3_^‐^‐responsive transcription (Liu et al., 2022a; Marchive et al., 2013; Figure 1). Interestingly, some members of the NRT1/NPF family have also been shown to transport additional substrates (Kanstrup et al., 2022). NRT1.1 transports auxin that mediates differential root growth depending on soil's NO_3_^‐^ supply (Krouk et al., 2010). Thus, NO_3_^‐^ influx competes with auxin import, the latter inhibiting root elongation and branching, to adjust root architecture to NO_3_^‐^ availability. Similarly, NPF5.12/TOB1 has been shown to transport NO_3_^‐^ and indole‐3‐butyric acid, an auxin precursor, to mediate cytokinin regulation of root architecture (Michniewicz et al., 2019). Recently, it has been discovered that sugars can also be substrates for NRT1/NPF activity. Thus, SUCROSE AND GLUCOSE CARRIER 1 (ZmSUGCAR1), a maize NRT1/NPF‐type transporter, is able to transport sucrose and glucose. Loss‐of‐function mutation of *ZmSUGCAR1* caused a significant decrease in sucrose and glucose content, provoking a reduction in kernel size. Moreover, it seems that this function is conserved in cereals since orthologues of ZmSUGCAR1 from wheat and sorghum displayed similar sugar transport activities (Yang et al., 2022).

While members of the NRT1 protein family serve mainly as low‐affinity transporters, the NRT2 protein family functions as high‐affinity transporters. The roots are the predominant site of expression for all *AtNRT2* genes and the responsivity and induced expression of different *NRT2* genes to changes in NO_3_^‐^ availability vary significantly. Thus, in *Arabidopsis*, NRT2.1, NRT2.2, NRT2.4, and NRT2.5 are mediators of high‐affinity NO_3_^‐^ uptake, although the latter two proteins function only under N starvation (Ruffel et al., 2011). The high‐affinity NO_3_^‐^ transporter NRT2.1 is the main transporter under low N conditions (Zhuo et al., 1999) and is transcriptionally and post‐transcriptionally regulated by C‐ and N‐derived metabolites, making it a central player in the integration of these signals. Thus, *NRT2.1* is induced both by N starvation and by light and sugars, suggesting that it mediates the coordination between N signalling and photosynthesis (Cerezo et al., 2001; Gansel et al., 2001; Lejay et al., 1999, 2003). Lateral root initiation is strongly repressed in Arabidopsis by the combination of high external sucrose and low external NO_3_^‐^ and NRT2.1 seems to act as a sensor for this C/N balance response in lateral root formation (Asim et al., 2020; Little et al., 2005; Zhang et al., 2007).

Finally, the glutamate receptor GLR1.1 has also an important role in C/N balance response. The plant glutamate‐like receptors (GLRs) are homologs of mammalian ionotropic glutamate receptors (iGluRs) and function in several processes related to plant growth, development, physiology and environmental stress responses (Weiland et al., 2015). Thus, the activities or transcript levels of several genes encoding enzymes related to C or N metabolism are reduced in plants defective in this gene. Interestingly, these plants also showed altered expression of several ABA biosynthetic and signalling genes together with enhanced drought tolerance, suggesting a role for this receptor in plant response to water stress (Kang et al., 2003, 2004). Further studies will elucidate how AtGLR1.1 integrates C/N ratios and stress response with plant development.

## 4 C/N SIGNALLING

**FIGURE 2:**
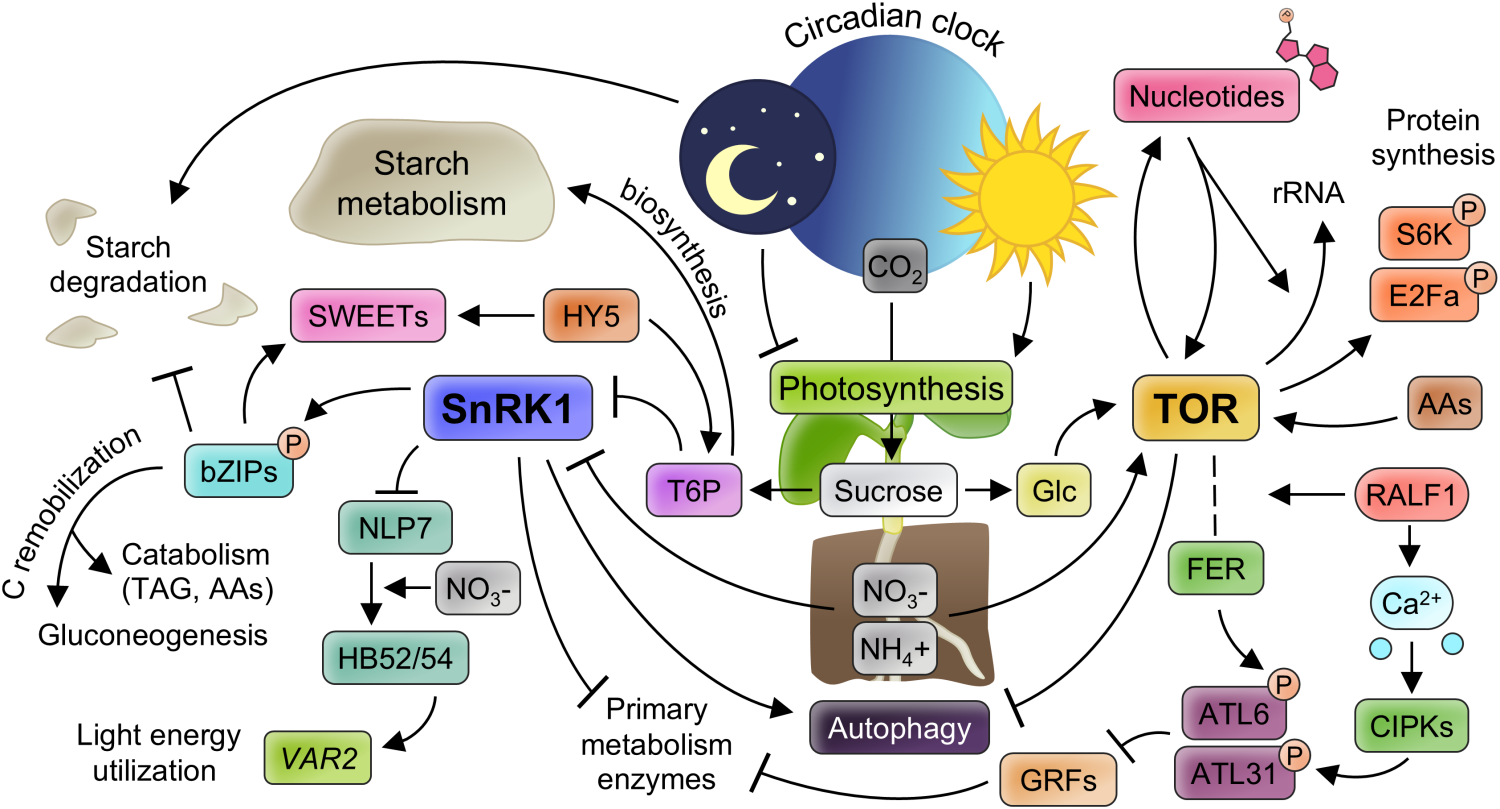
Graphic representation of SnRK1 and TOR regulatory networks and their interaction with C/N signalling pathways. The left side of the figure depicts the relationship between SnRK1 and sugar‐related transporters and signalling proteins, as well as NO_3_^‐^ sensing. The right side represents links between TOR, protein and nucleotide synthesis, and regulators of C/N balance. Both sides are interconnected by C and N‐uptake/assimilation processes shown in the middle of the figure. The formation of complexes is indicated by dotted lines. Glc: Glucose. AAs: Amino Acids.

TOR and SnRK1 are two kinase complexes conserved among eukaryotes that play antagonistic key roles in nutrient signalling (Artins et al., 2024; Artins & Caldana, 2022; Dobrenel et al., 2016; Fichtner et al., 2021b; McCready et al., 2020; Peixoto & Baena‐Gonzalez, 2022; Ryabova et al., 2019; Wu et al., 2019). The TOR pathway responds to energy, nutrients, environmental cues and hormones and is activated under nutrient sufficiency to adjust growth according to available resources. Important enzymes involved in the metabolism of C and N compounds are putatively phosphorylated by TOR (Van Leene et al., 2019). It is worth mentioning that, while TOR expression in mammals is detected in all cells (Chiu et al., 1994), TOR expression in plants is limited to the zones where cell proliferation is coupled to cell growth, mostly cytosolic, such as embryo, endosperm and primary apical and root meristems (Menand et al., 2002). Particularly relevant for this growth is the glucose‐TOR signalling that integrates light, sugar availability (photosynthetic activity and C‐assimilation) and auxin levels to control meristem activation in roots and shoots (Chen et al., 2018; Li et al., 2017; Pfeiffer et al., 2016; Xiong et al., 2013; Figure 2). The absence of TOR expression in differentiated mature cells, whose growth process is mostly related to vacuole and cell‐wall expansion instead of cytosolic, points to the existence of a different nutrient sensing mechanism (Menand et al., 2002). In addition to its activation by sugars (sucrose and glucose), TOR also couples changes in sugar levels to the metabolism of N‐compounds. For instance, studies in Chlamydomonas showed that impaired CO_2_ fixation inhibits TOR activity, which correlates with a gradual decrease of most amino acids (Mallen‐Ponce et al., 2022). Starch synthesis is essential to keep photosynthetic electron transport capacity and, when impaired, causes a drop in photosynthetic function and a redirection of photosynthetically fixed C (Saroussi et al., 2019). Remarkably, a mutant unable to synthesize starch had higher TOR activity, probably due to the concomitant elevation of Gln concentration (Mallen‐Ponce et al., 2022), an amino acid known to activate TOR (Gonzalez & Hall, 2017). TOR activity can also be altered by inorganic and organic N‐signals. In leaf primordium of Arabidopsis under N‐deficiency, TOR is activated when NO_3_^‐^ and NH_4_ are supplied, independently of their assimilation or glucose‐energy and hormone signalling pathways (Liu et al., 2021; Figure 2). In the same study, it was found that 15 out of the 20 proteinogenic amino acids were able to activate TOR, with several of them being derived from specific C‐compounds produced in glycolysis (pyruvate) and TCA (oxalacetate). A previous study showed the ability of certain amino acids to upregulate TOR signalling and impact homeostasis and organization of actin cytoskeleton and endomembranes (Cao et al., 2019; Figure 2). In line with the above, TOR activates growth in response to C and N signals through the promotion of protein synthesis by phosphorylation of the E2Fa TF and the S6K ribosomal protein kinase (Li et al., 2017; Henriques et al., 2013; Schepetilnikov et al., 2013; Xiong et al., 2013; Figure 2). Genetic or chemical downregulation of components of TOR leads to a fast increase in the levels of amino acid levels in plants (Salem et al., 2017), which could be explained by the inability to use them efficiently for protein synthesis. TOR also promotes protein translation by coordinating ribosomal RNA synthesis with nucleotide availability (C‐ and N‐containing compounds) and its activity is inhibited when nucleotide biosynthesis is disrupted (Busche et al., 2021; Figure 2).

It is well‐known that energy and C starvation activate SnRK1 to phosphorylate and inactivate TOR. In addition, SnRK1 also modulates the phosphorylation status of about 500 proteins (Cho et al., 2016; Li et al., 2020b; Nukarinen et al., 2016; Van Leene et al., 2022), affecting the activity of key enzymes for N, C or fatty acid metabolism (Harthill et al., 2006; Kulma et al., 2004). For example, SnRK1 also induces autophagy processes through differential phosphorylation of ATG (AuTophaGy) proteins, and acts upstream of the negative regulation exerted by TOR on the same processes (Chen et al., 2017; Huang et al., 2019; Liu & Bassham, 2010; Mugume et al., 2020; Pu et al., 2017; Soto‐Burgos & Bassham, 2017; Figure 2). Activation of SnRK1 also triggers its translocation to the nucleus to promote transcription of over 1,000 genes destined to reduce energy consumption (Baena‐González et al., 2007; Ramon et al., 2019). Part of this regulation is exerted by the phosphorylation of bZIP63 and putatively other C/S1 bZIP TFs (Baena‐González et al., 2007; Baena‐González & Sheen, 2008; Mair et al., 2015). For instance, SnRK1 promotes the catabolism of triacylglycerols (TAGs) and amino acids during seedling establishment to fuel gluconeogenesis through bZIP63 (Henninger et al., 2022; Figure 2). SnRK1 has also been proposed to couple energy metabolism with growth under diurnal cycles through the clock‐regulated bZIP63 by preventing the acceleration of starch degradation at the end of the night (Viana et al., 2021). Interestingly, SnRK1 signalling is higher at the end of the night, coinciding with the lowest levels of T6P (Peixoto et al., 2021). As mentioned above, T6P is a proxy for sucrose levels and promotes starch biosynthesis, interplaying antagonistically with SnRK1 to control sucrose homeostasis (Figure 2). A clear connection between the role of SnRK1 on the control of sucrose levels and N metabolism comes from maize. Lower activity of SnRK1 under increased sucrose levels has been linked to the translocation of the O2 TF to the nucleus to promote zein synthesis, the main N‐storage compound in maize seeds (Li et al., 2020a). However, when SnRK1 is artificially overexpressed in Arabidopsis plants grown under elevated glucose/sucrose levels, growth arrest is promoted due to enhanced phosphorylation and activity of SnRK1 (Jossier et al., 2009). Finally, SnRK1 activity is also under the control of N‐signals given that NO_3_^‐^ promotes degradation of the catalytic subunit of the SnRK1 complex (KIN10), while NO_3_^‐^ depletion induces KIN10 accumulation (Wang et al., 2022; Figure 2). Whether this mechanism depends on C/N ratios remains to be elucidated.

Interestingly, KIN10 phosphorylates NLP7, the NO_3_^‐^ sensor, to promote its cytoplasmic localization and degradation (Wang et al., 2022). Thus, N‐sufficiency favours NLP7 nuclear localization and signalling, both of which would be counteracted by SnRK1 (KIN10) under N‐deficiency. NLP7 was also found to orchestrate a NO_3_^‐^‐activated pathway that resulted in improved light energy utilization under high light and low N conditions. This pathway enhances the expression of a subunit of the chloroplast *VAR2* protease by a cascade of direct transcriptional activations. These activations are carried out chronologically by NLP7 and the HB52/54 TFs, which facilitate the removal of photodamaged‐thylakoid membrane proteins (Ariga et al., 2022; Figure 2). In this sense, the chloroplast is an important organelle for nutrient homeostasis and the location of the PII protein, a highly conserved signal transduction regulator of C/N interactions. In plants, the *PII* gene is encoded by the nuclear genome and targeted to the chloroplast, and its transcription is positively regulated by light and sucrose and negatively by amino acids (Hsieh et al., 1998; Nunes‐Nesi et al., 2010). Although its role in C and N metabolism may not be as important as in bacterial systems, several results indicate that PII connects C/N pathways in plants. For instance, PII regulates the first committed step of Arg biosynthesis by interacting with a chloroplastic kinase (Chen et al., 2006; Ferrario‐Méry et al., 2006) and PII mutants show reduced accumulation of Arg and Gln, and higher levels of starch and sugars when grown under NH_4_^+^ (Ferrario‐Méry et al., 2005, 2008). In addition, PII seems to inhibit lipid biosynthesis since it interacts with and inhibits the first enzyme of fatty acid synthesis in plastids (ACCase), an inhibition reverted by 2‐OG (Feria Bourrellier et al., 2010). Interestingly, PII expression is upregulated during early seed development by the WRI1 TF, a master positive regulator of glycolysis and seed‐storage oil synthesis. This effect might enable plants to fine‐tune fatty acid content (Baud et al., 2010) and to channel some of the C‐skeletons towards N‐compounds.

An aspect not very well studied in C/N responses is the possible existence of organ‐type or/and developmental‐specific signalling mechanisms. For instance, another relevant study on this matter revealed that C‐ and N‐acquisition is coordinated between roots and shoots by the mobile HY5 TF (Chen et al., 2016). Upon illumination of the shoot, HY5 stimulates NO_3_^‐^ uptake by moving from shoot to root through the phloem, where it activates its own expression and *NRT2.1* expression. This effect is enhanced by sucrose/glucose via an unknown mechanism, probably to keep balanced C/N ratios. In addition, HY5 also affects both sucrose metabolism and shoot‐to‐root transport by promoting the expression of T6P synthase (*TPS1*) and *SWEET11/12* sucrose efflux transporters (Chen et al., 2016; Figures 1 and 2). These *SWEET* genes are also among those directly transactivated by several bZIP TFs that were found to promote apical dominance and restrict lateral organ formation by favouring redistribution of C‐ and N‐compounds from source leaves to apical organs (Kreisz et al., 2024). Loss of S_1_‐type bZIPs function produced reduced leaf sugar export to the apoplast for long‐distance transport to distal sinks, which increased the symplastic sugar flux to proximal sinks (increased C/N ratios in rosette leaves). Simultaneously, the lack of glutaminase transactivation rendered increased levels of Gln and Asn in seedling roots and increased Gln in the shoot just before bolting (Kreisz et al., 2024). These and previous findings position the S_1_‐type bZIPs as key players in C/N and energy responses with a wide regulatory spectrum. For instance, under low‐energy conditions, they promote the degradation of proline and branched amino acids to feed C skeletons into alternative respiratory pathways for ATP production (Pedrotti et al., 2018; Figure 2). They also heterodimerize with the C‐type bZIP63 to mediate key aspects of SnRK1 functions (Ehlert et al., 2006; Henninger et al., 2022; Muralidhara et al., 2021; Mair et al., 2015). The above results also point to the possible existence of regulatory and molecular interactions between HY5 and S_1_‐type bZIPs, which may have been hidden so far due to the genetic redundancy of the bZIPs.

14‐3‐3 proteins (also and hereafter called General Regulatory Factors, GRFs) can be found in different subcellular locations and have extensive regulatory effects on C and N metabolism in plants. GRF proteins generally interact with phosphorylated target proteins to regulate their functions by different mechanisms (Jaspert et al., 2011; Zhao et al., 2021). Several of the GRF target proteins are enzymes of the C and N metabolism (Comparot et al., 2003) as relevant as NO_3_^‐^ reductase (Bachmann et al., 1996), sucrose‐phosphate synthase (Moorhead et al., 1999; Toroser et al., 1998), starch synthase III family members (Sehnke et al., 2001), trehalose‐6‐phosphate synthase or cytosolic and plastidic forms of glutamine synthetase (Moorhead et al., 1999; Figure 2). GRF proteins also seem to be directly linked to energy status since 5´‐AMP has been shown to reduce the interaction between GRF and their targets (Athwal et al., 1998). Unlike animals, plants use mostly protons and H^+^‐ATPase pumps to transduce chemical and environmental signals and support a range of physiological functions (Sze et al., 1999). Interestingly, several GRFs have been shown to interact with and regulate chloroplast, mitochondrial and plasma membrane ATPases (Bunney et al., 2001; Jahn et al., 1997; Sze et al., 1999). Several lines of evidence point to the existence of functional redundancy in the GRF family and their differential regulation of metabolic enzymes. Thus, Arabidopsis GRF1, GRF3 and GRF8 proteins showed overlapping and specific interactions with 150‐200 proteins. Furthermore, when the activities of enzymes targeted by GRF were compared by using gain‐ and loss‐of‐function mutants, results confirmed the existence of partial functional redundancy in this family of regulators (Shin et al., 2011). In a separate study, overexpression of Arabidopsis GRFs triggered decreased levels of sugars, malate, citrate and N‐containing‐compounds as well as a reduction in the activities of known GRF‐interacting‐enzymes such as the TCA cycle enzymes isocitrate dehydrogenase and malate dehydrogenase (Diaz et al., 2011). Additional evidence associating their negative regulation on the activity of several C‐ and N‐biosynthetic enzymes was obtained from the downregulation of several GRFs in potato and Arabidopsis (Sehnke et al., 2001; Świȩdrych et al., 2002; Zuk et al., 2003, 2005). In addition to regulating the activity of their target enzymes, molecular evidence of proteolytic degradation of GRF target proteins has been obtained in *Medicago truncatula* (Lima et al., 2006) and under sugar starvation in Arabidopsis (Cotelle et al., 2000).

The stress caused by high C/N induces the accumulation of GRF proteins, and hypersensitivity to this stress has been observed when ectopically overexpressing *GRF1* (Sato et al., 2011). High C/N also induces *ATL31/CNI1* expression, an E3‐ligase that interacts with GRF proteins at the membrane and mediates their degradation by the proteasome. This mechanism allows modulation and fine‐tuning of GRF activity to control the response and reduce stress sensitivity to high C/N stress (Aoyama et al., 2014; Sato et al., 2011; Yasuda et al., 2014). ATL31 ligase, as well as it closest relative ATL6, are under the positive control of a group of kinases. CIPK7, 12 and 14 phosphorylate and stabilize ATL31 and both activities are required for the interaction and degradation of GRF target proteins (Yasuda et al., 2017). Likewise, the FER kinase mediates ATL6 phosphorylation to enhance the interaction between ATL6 and its target GRF proteins at the membrane (Xu et al., 2019). Not surprisingly, loss‐of‐function in these kinases (CIPKs or FER) causes hypersensitivity to high C/N ratios (Xu et al., 2019; Yasuda et al., 2017). It was also found that the direct phosphorylation of ATL31 by CIPK14 is preceded by the interaction of the kinase with the CBL8 Ca^2+^ sensor at the plasma membrane in a Ca^2+^‐dependent manner (Batistič et al., 2010; Yasuda et al., 2017). This event highlights the existence and relevance of non‐nuclear control mechanisms for C/N responses and growth. In support of this evidence, FER is also a membrane receptor kinase that mediates C/N responses and has additional roles in the control of growth and stress responses (Liu et al., 2023; Song et al., 2022; Tang et al., 2022; Yang et al., 2015). FER is bound and activated by RALF1, a secreted peptide that induces a rapid increase in cytoplasmic Ca_2_^+^ (Haruta et al., 2014). FER, in turn, interacts with and phosphorylates ATL6 at the membrane to reduce GRF levels and sensitivity to high C/N (Xu et al., 2019). Moreover, RALF1 increases TOR activation by enhancing FER‐TOR interactions, promoting true leaf growth under N‐deficient conditions in Arabidopsis (Song et al., 2022). All these results underscore the existence of different tissue‐specific outcomes of the RALF1‐FER interaction and the impact of several subcellular compartments and developmental stages to signal the C/N status (Figure 2).

## 5 PHYTOHORMONES IN C/N SIGNALLING

Efficient and rapid communication throughout the plant body is key for successful coordination of growth and function in multiple organs with different nutrient needs. This requires both local and long‐distance signalling for the integration of environmental, nutritional and developmental signals (Yu et al., 2015; Wheeldon & Bennett, 2021). In recent years, phytohormones have been increasingly linked to energy and nutrient homeostasis, particularly C and N metabolism (Ahmad et al., 2023; Fabregas & Fernie, 2021, 2022; Fichtner et al., 2021b; Jamsheer et al., 2019; Sakr et al., 2018). In some cases, the impact of high C availability on plant development and hormone signalling seems to be dependent on N levels (Cho et al., 2010; Lu et al., 2015b; Pourtau et al., 2004). This section will highlight their emerging roles in C/N nutritional balance by focusing on the links between hormone pathways, the kinase complexes TOR and SnRK1, and C and N signalling.

### 5.1 Abscisic acid

**FIGURE 3:**
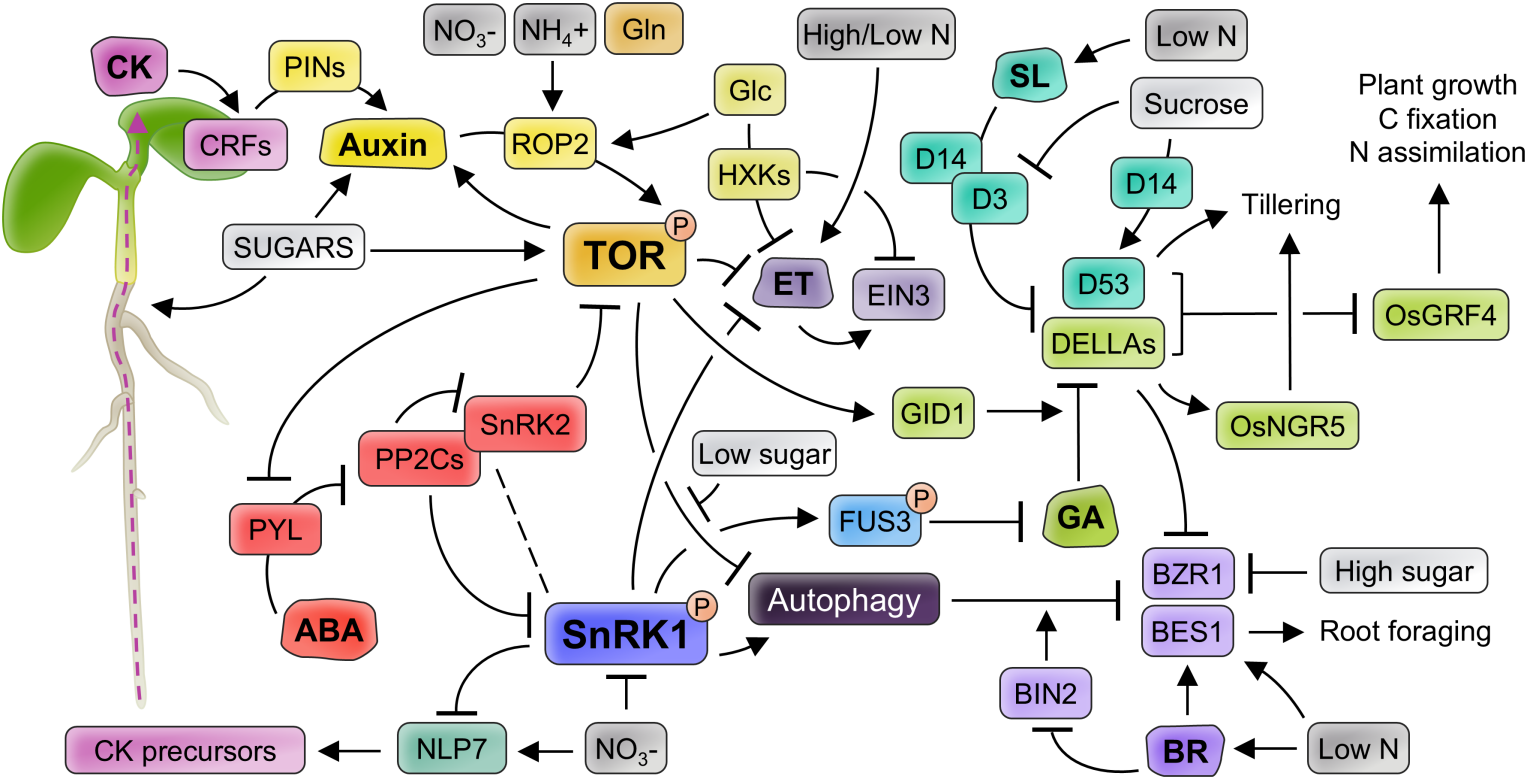
Overview of phytohormones main signalling mechanisms linked to C/N balance. The different hormonal and signalling components are shown from left to right: Cytokinins (CK), abscisic acid (ABA), auxins, ethylene (ET), strigolactones (SL), gibberellins (GA) and brassinosteroids (BR). The elements of each hormone's network are shown in the same colour. The formation of complexes is indicated by dotted lines. Glc: Glucose.

ABA regulates growth at multiple developmental stages throughout the life of the plant and a wide range of physiological processes in response to stress signals and changing environments (Chen et al., 2020; Parwez et al., 2022). The core of ABA signalling relies on the hormone recognition by intracellular receptors (PYR1/PYL/RCAR), triggering a conformational change to allow binding to PP2Cs phosphatases, thus releasing their inhibition on SnRK2 protein kinases (Umezawa et al., 2010; Figure 3). SnRK2s activate multiple physiological responses by phosphorylating downstream substrates such ABF TFs (Umezawa et al., 2013; Wang et al., 2013).

Over the years, various links have been established between ABA and the key metabolic regulators SnRK1 and TOR. Genetic, molecular and physiological analyses have revealed the involvement of SnRK1 in both sugar and ABA signalling pathways (Jossier et al., 2009), including largely overlapping transcriptional responses (Carianopol et al., 2020). Thus, under optimal conditions (low ABA), PP2C‐SnRK2 complexes directly bind and inhibit SnRK1 (Belda‐Palazón et al., 2020, 2022; Rodrigues et al., 2013), while stress scenarios would increase ABA levels and allow the coordinated activation of both ABA and SnRK1 pathways (Figure 3). A direct link between SnRK1 and ABA perception has also been identified since the overexpression of an ABA receptor (*RCAR6*) leads to the activation of both SnRK1 and SnRK2 (Yoshida et al., 2024). Downstream of ABA reception, SnRK1 activity is known to regulate ABA‐related TFs, such as FUS3 (Chan et al., 2017; Gazzarrini et al., 2004; Tsai & Gazzarrini, 2012) or ABF2 (Rodrigues et al., 2013). On the other hand, reciprocal regulation of TOR and SnRK2 has been described. Thus, TOR phosphorylates ABA receptors to prevent activation of SnRK2 under favourable environmental conditions, whereas under stress ABA‐activated SnRK2s trigger the dissociation and inhibition of the TOR complex (Wang et al., 2018a; Figure 3).

C and N signalling networks also interact with ABA at different intersection points. The genetic screen of mutagenized Arabidopsis plants for altered sugar sensitivity has often led to the identification of ABA‐deficient or insensitive mutants (Fabregas and Fernie 2021). On the other hand, several lines of evidence have linked root adaptation to NO_3_^‐^, a response that couples lateral root growth to NO_3_^‐^ levels with ABA signalling (Contreras‐López et al., 2022; Kim et al., 2004; Léran et al., 2015; Ondzighi‐Assoume et al., 2016; Rodrigues et al., 2013; Signora et al., 2001). The ABI1 phosphatase provides a direct crosstalk between C/N balance and ABA signalling pathway. *ABI1* overexpression produces a tolerance phenotype under extremely high C/N ratios, accompanied by altered expression of ABA and SnRK1s‐related genes (Lu et al., 2015a). The positive and master regulators of ABA signalling, ABI4 and ABI5, have also been linked to glucose and NO_3_^‐^ metabolism (Signora et al., 2001; Fabregas and Fernie 2021). ABI5 mediates the acclimation of Arabidopsis plants to low CO_2_ availability downstream of the phosphoenolpyruvate carboxylase PPC2, a response that requires the regulation of C and N metabolism (You et al., 2020). ABI4 act upstream of the transcription factor CHO1, which acts as a node of hormonal and nutritional signalling and regulates seedling growth under excess supply of glucose and NO_3_^‐^ (Yamagishi et al., 2009). Finally, ABI4 and ABI5 act synergistically in the activation of the diacylglycerol acyltransferase *DGAT1* expression, the rate‐limiting enzyme in TAG biosynthesis, in Arabidopsis seedlings under abiotic stresses, N deficiency and C/N imbalance (Yang et al., 2011; Kong et al., 2013).

### 5.2 Auxins

Auxin is an essential hormone that integrates endogenous developmental signals and exogenous environmental cues to coordinate the growth and development of spatially distant organs (Casanova‐Sáez et al., 2021; Zhao, 2018). Over the years, accumulating evidence has demonstrated that the auxin pathway interacts with and coordinates C and N metabolism to shape the plant architecture at different growth stages and under dynamic environmental conditions (Fichtner et al., 2020; Gutiérrez et al., 2007). It has been shown that different soluble sugars derived from photosynthesis regulate auxin biosynthesis via HXK‐dependent and independent signalling, as well as via PIF TFs (Lilley et al., 2012; Sairanen et al., 2012; Figure 3). Auxins, in turn, fine‐tune sugar metabolism and source‐sink C partitioning (Fichtner et al., 2020; Mishra et al., 2022). At the molecular level, in both shoot and root apical meristems, systemic glucose and enhanced auxin accumulation activates the ROP2 GTPase, which physically interacts with and triggers TOR phosphorylation and activation, upregulating cell cycle genes and DNA synthesis (Li et al., 2017; Schepetilnikov et al., 2013, 2017; Xiong et al., 2013; Figure 3). However, in the root elongation zone, glucose‐activated TOR phosphorylates and stabilizes PIN2, an auxin efflux facilitator, to maintain the low auxin levels required for cell expansion and root patterning (Yuan et al., 2020). As with glucose, ROP2 has been described to integrate the presence of auxin and primary N signals (NO_3_^‐^, NH_4_^+^ and Gln) to promote cell division and true leaf development through TOR activation (Li et al., 2017; Liu et al., 2021; Schepetilnikov et al., 2017; Figure 3). In addition, TOR has been recently described to promote the translation of the auxin‐induced ARF7, ARF19 and LBD16 TFs, essential players in root branching, in a sugar‐dependent manner (Stitz et al., 2023). How this C‐mediated regulation by TOR is affected by N‐availability remains to be determined. Consequently, disruption of the TOR pathway leads to repression of auxin biosynthesis, transport and signalling, resulting in impaired auxin‐mediated plant growth and stress responses (Deng et al., 2016, 2017; Dong et al., 2015; Li et al., 2017; Schepetilnikov et al., 2013). On the other side, some links between NO_3_^‐^‐supply and auxin translocation and accumulation have been reported. At low N availability, NRT1.1 prevents auxin accumulation by lowering its biosynthesis and transport to the tip of the lateral root primordia (LRP), thereby contributing to slowing down LRP development and emergence in NO_3_^‐^‐poorer areas (Krouk et al., 2010; Maghiaoui et al. 2020; Mounier et al., 2014). The integration of N signals into auxin signalling has also been described in combination with cytokinins (CKs). NLP7 coordinates shoot growth in response to NO_3_^‐^ supply via stimulating CKs biosynthesis and transport from root to shoot. There, the enhanced expression of CRFs directly regulates *PIN* transporters, promoting the accumulation and, thereby, stimulating shoot growth (Asim et al., 2020; Abualia et al., 2022; Kumar et al., 2023; Figure 3). Collectively, these findings reinforce that auxin accumulation, distribution and function in the plant is vastly controlled by sugar metabolism and N availability.

### 5.3 Brassinosteroids

It is well established that brassinosteroids (BRs) play critical roles in plant growth and developmental processes but also responses to environmental stimuli (Nolan et al., 2020). At the first steps of their signalling cascade, BRs binding induces the phosphorylation of the receptor kinase BRI1, which triggers a phosphorylation cascade that results in the inactivation of the BIN2 kinase and subsequent activation of the BES1/BZR1 TFs, key positive regulators of downstream BR‐responsive genes (Nolan et al., 2020; Han et al., 2023). In recent years, BR signalling research has led to the characterization of crosstalk between these core components, C metabolism, N availability and TOR signalling (Han et al., 2023). BRs are required for normal plastid development and CO_2_ assimilation (Schröder et al., 2014) as well as for TCA cycle activity, starch accumulation and biomass production (Siddiqui et al., 2018). Conversely, C availability modulates BR signalling in a dose‐dependent manner (Han et al., 2023; Zhang et al., 2015). Thus, although proper concentration of sugar activates BR signalling, the pathway is inhibited under sugar starvation or surplus, mainly by reducing BZR1/BES1 activity (Han et al., 2023). Sugar starvation causes BZR1/BES1 degradation via selective autophagy (Nolan et al., 2017; Wang et al., 2021b; Zhang et al., 2016), a pathway enhanced by BIN2 and SnRK1, and negatively regulated by TOR (Liao et al., 2023; Soto‐Burgos & Bassham, 2017; Figure 3). On the other hand, high concentrations of sucrose also inhibit BR‐signalling by promoting the phosphorylation and cytosolic retention of BZR1 (Hao et al., 2016; Zhang et al., 2021). Moreover, BR signalling is also highly dependent on the availability of N in the environment (Han et al., 2023). Mild N deficiency induces BR biosynthesis (Jia et al., 2020) and also increases BR signalling through the accumulation and activation of BES1. Activated BES1 suppresses the expression and DNA binding ability of the LBD37/38/39 TFs, thus enhancing the expression of genes that stimulate foraging response under low N conditions (Chai et al., 2022; Figure 3).

### 5.4 Cytokinins

CKs are hormones that participate in the regulation of plant growth, nutritional signalling, and responses to abiotic and biotic stresses (Li et al., 2021b). CKs signalling participates in local and long‐distance shoot–root communication, and the adjustment of plant architecture in response to C metabolism and external and internal N status. Both signals have been shown to induce CK synthesis and accumulation via the upregulation of *IPT* genes (Wang et al., 2021a; Abualia et al., 2023). On one hand, photosynthetically generated sugars induce the biosynthesis and transportation of CK precursors from the root to the shoot to activate CK signalling and stimulate plant growth (Kiba et al., 2019; Figure 3). In return, CKs can also influence C metabolism and many CK response mutants display altered sensitivity to sugars (Radchuk et al., 2023; Wang et al., 2021a). Also, CKs are implicated in sugar‐induced anthocyanin accumulation in Arabidopsis leaves (Sakr et al., 2018). As mentioned, CKs function as a root‐to‐shoot long‐distance N status signal that regulates both N‐related metabolism and transport to adjust plant growth to N availability. In roots, NO_3_^‐^ supply induces CKs biosynthesis and translocation through the xylem via NLP7. Then, CK accumulation promotes shoot growth through increasing expression of auxin efflux carriers and induces a systemic downstream signal that regulates NO_3_^‐^ transporter expression in roots (Abualia et al., 2022; Landrein et al., 2018; Poitout et al., 2018; Figure 3). Consistently, numerous components of the CK signal transduction pathway, including type‐A ARR as well as CRF TFs, have been identified as NO_3_^‐^‐responsive genes (Abualia et al., 2023). Although CK‐dependent coordinated regulation of C and N metabolism has already been observed (Reguera et al., 2013), the role of this phytohormone in the combined regulation of C/N balance in the plant needs to be elucidated.

### 5.5 Gibberellins

GAs and C metabolism interplay in the control of photomorphogenesis processes like cotyledon opening and the regulation of light‐responsive gene expression (Feng et al., 2008). Modulation of GA levels in response to light and C metabolism extends to environmental CO_2_ (Ribeiro et al., 2012), circadian rhythms, as they track daily fluctuations in sugar levels (Paparelli et al., 2013), and the TCA cycle, with the intermediate 2‐OG (2‐oxoglutarate) controlling the rate of biosynthesis of GAs and amino acids (Araújo et al., 2014). GAs are also involved in the induction of the anthocyanin biosynthesis pathway, which is dependent on the sucrose stabilisation of DELLA proteins (Li et al., 2014). DELLAs are nuclear proteins lacking a DNA‐binding domain that negatively regulate GA responses through interaction with other TFs and are targeted for degradation in the presence of GA and the GA receptor GID1 (Daviere & Achard, 2013). Regarding N metabolism, the only DELLA protein in rice (SLR1) functions as a pivotal factor in the integration of GA signalling pathways and N deficiency‐induced adaptations on plant architecture. On one hand, OsGRF4 TF activity is inhibited by DELLAs, thus balancing N assimilation, C fixation and plant growth. Increasing the abundance of OsGRF4 tips the GRF‐DELLA balance to favour GRF4, which increases growth and N‐use efficiency (NUE; Li et al., 2018; Figure 3) On the other hand, DELLA accumulation increases the stability of OsNGR5, a protein that promotes rice tillering in the presence of N, by competitively binding to the gibberellin receptor GID1 (Wu et al., 2020; Figure 3). In addition, Camut et al. (2021) revealed that NO_3_^‐^ enhances the accumulation of bioactive GAs in both Arabidopsis and wheat, thus reducing the abundance of DELLAs and promoting plant growth. Finally, a few reports suggest the interaction of GA signalling with TOR and SnRK1. Zhang et al. (2018) revealed that disruption of the major RAPTOR isoform, RAPTOR1B, leads to a decrease in transcript levels of the GA receptor *GID1*. On the other hand, SnRK1 can repress GA biosynthesis through FUS3 activation (Curaba et al., 2004; Gazzarrini et al., 2004; Figure 3).

### 5.6 Other phytohormones

Several other phytohormones have been related to N and C pathways regulation but their direct contribution to the control of plant response to C/N balance has not been clearly established yet. For instance, high levels of strigolactones (SLs) in the root modify its architecture to increase their coverage in the surrounding soil and the root exudates that promote plant–microbe symbiotic interactions, which together improve mineral nutrients and water uptake (Barbier et al., 2023; Dun et al., 2023; Kelly et al., 2023; Sun et al., 2022). SLs are also transported through the xylem from roots to shoots, where they can act as a signal to coordinate shoot branching with the nutrient status of the roots (Dun et al., 2023; Kelly et al., 2023). Moreover, Sun et al. (2023) revealed a crosstalk between the SL and GA pathways in response to changes in N availability in rice. Under high N supply, the D53 and SLR1‐DELLA proteins, both key repressors of the SL and GA signalling pathways, respectively, bind to the GRF4 TF, reducing the activation of N metabolism‐associated genes. This regulation is reverted under N limitation by SL‐mediated promoted degradation of the D53 and SLR1 proteins (Figure 3). SLs also mediate C metabolism and sugar signalling in early seedling development (Li et al., 2016) and shoot branching. In this respect, SL negative effect over tillering in rice involves the formation of D3‐D14‐D53 complex and subsequent degradation of D14 and D53. This mechanism is dependent on sucrose levels, which can alleviate SL‐mediated inhibition of bud outgrowth through D3 repression and the triggering of a D14‐dependent induction of D53 accumulation (Patil et al., 2022; Figure 3). This crosstalk and the role of MAX2 (D3 orthologue) have also been described in Arabidopsis (Bennett et al., 2006; Bertheloot et al., 2020).

Finally, genetic and phenotypic approaches have uncovered an antagonistic interplay between sugars and ethylene pathways (Sakr et al., 2018), where glucose sensors HXK1 and HKL1 have been described as direct regulators of glucose‐dependent transcriptional repression of some ethylene biosynthesis genes. Meanwhile, HKL1 can also act as a positive effector of ethylene responses (Karve et al., 2012). EIN3 TF, whose stability is enhanced by ethylene and reduced by glucose, is also a key regulator of this interaction (Yanagisawa et al., 2003; Figure 3). The relationship between ethylene and C signalling has been described in developmental processes like the dark‐to‐light transition of seedlings (Brenya et al., 2023; Zhong et al., 2014), adaptation to high atmospheric CO_2_ (Smet et al., 2020) or the long‐distance transport of sucrose from source leaves to sink organs (Tong et al., 2022). Interestingly, ethylene biosynthesis and signalling are negatively regulated by the master regulators TOR (Dong et al., 2015; Fu et al., 2021; Zhuo et al., 2020; Figure 3) and SnRK1 (Kim et al., 2017; Lumba et al., 2012). As with sugars, ethylene also plays an essential role in plant responses to mineral nutrient availability, including N. In summary, the exposition to sub or supra‐optimal N status stimulates ethylene biosynthesis and signalling in the plant (Figure 3). Then, induced ethylene mediates a range of adaptive responses that will re‐model root architecture and modulate the overall assimilation, translocation and accumulation of N (Khan et al., 2015; Ma et al., 2023b).

## 6 CONCLUSION

In this review, we have focused on aspects of the C and N metabolism and signalling that may be underlying mechanisms responsible for maintaining C/N balance in plants and provided a comprehensive, as well as reasonably summarized picture of their features. We still have numerous gaps in our understanding of the molecular and hormonal control of this important process, as well as their connection with plant development and stress responses. Results from these studies will open new avenues to improve crop yield by enhancing their ability to cope with nutrient imbalance and stress scenarios.

## AUTHOR CONTRIBUTIONS

All authors participated in literature search, figure design and manuscript writing. IF‐P drew the figures. M.P. and L.O‐S planned and edited the manuscript. All the authors have read and approved the final version of the manuscript.

## Supporting information


**Table S1.** List of complete gene names in alphabetical order according to their acronyms.

## Data Availability

N/A
