# Supplementary material for: Signalling and regulation of plant development by carbon/nitrogen balance
Source: Physiol Plant. 2025 Apr 23;177(2):e70228. doi: 10.1111/ppl.70228 (PMC12018728; doi:10.1111/ppl.70228)
Supplement: Supplementary file 1 — Table S1. List of complete gene names in alphabetical order according to their acronyms. [file PPL-177-e70228-s001.docx]

***Table S1****. List of complete gene names in alphabetical order according to their acronyms*

| **Abbreviation/**  **gene name** | **Complete name** | **Gene type** |
| --- | --- | --- |
| ABFs | ABA-Responsive Element Binding Factors | bZIP TF |
| ABI1 | Abscisic-acid insensitive 1 | Type 2C phosphatase |
| ABI2 | Abscisic acid insensitive 2 | Type 2C phosphatase |
| ABI4 | Abscisic-acid insensitive 4 | ERF TF |
| ABI5 | Abscisic-acid insensitive 5 | bZIP TF |
| ACCase | Acetyl-CoA carboxylase | Fatty acid synthesis enzyme |
| ARE4 | Abc1-1 rescued 4 | MYB TF |
| ARF7 | Auxin response factor 7 | ARF TF |
| ARF19 | Auxin response factor 19 | ARF TF |
| ARRs | Arabidopsis response regulators (TFs) | CK negative regulators |
| ATG | Autophagy protein |  |
| ATL6 | Arabidopsis tóxico en levaduras 6 | Ring-H2 type ubiquitin ligase |
| ATL31/CNI1 | Arabidopsis tóxico en levaduras 31/ carbon/nitrogen insensitive 1-dominant | Ring-H2 type ubiquitin ligase |
| BES1 | BRI1 EMS SUPPRESSOR 1 | bHLH TF |
| BIN2 | Brassinosteroid insensitive 2 | Kinase |
| BRI1 | Brassinosterioid insensitive 1 | Plasma membrane receptor kinase |
| bZIP11 | Basic leucine zipper 11 | bZIP TF |
| bZIP63 | Basic leucine zipper 63 | bZIP TF |
| BZR1 | BRASSINAZOLE RESISTANT 1 | bHLH TF |
| CBC1/CBC2 | Convergence of blue light and CO_2_ | Raf kinase |
| CBL8 | Calcineurin B-like protein 8 | Ca^+2^ sensor |
| CHO1 | CHOTTO1 | AP2 TF |
| CIPK7 | CBL-interacting serine/threonine-protein kinase 7 | Serine/threonine-protein kinase |
| CIPK12 | CBL-interacting serine/threonine-protein kinase 12 | Serine/threonine-protein kinase |
| CIPK14 | CBL-interacting serine/threonine-protein kinase 14 | Serine/threonine-protein kinase |
| CIPK23 | CBL-interacting protein kinase 23 | Serine/threonine-protein kinase |
| CNGC15 | Cyclic nucleotide-gated channel protein 15 | Voltage-gated ion channel superfamily |
| CPK10/28/30/32 | Ca^+2^-dependent protein kinases 10/28/30/32 | Ca^+2^-dependent protein kinases |
| CRFs | Cytokinin Response Factors (TFs) | CK positive regulators |
| D3 | DWARF 3 | F-box protein |
| D14 | DWARF 14 | SL receptor |
| D53 | DWARF 53 | SL signalling repressor |
| DGAT1 | Diacylglycerol acyltransferase 1 | TAG biosynthesis |
| E2Fa | - | TF |
| EIN3 | Ethylene Insensitive 3 | TF |
| FER | Feronia | Receptor-like protein kinase |
| FINS1 | Fructose insensitive1 | Fructose 1,6-bisphosphatase |
| FUS3 | Fusca 3 | B3 TF |
| GID1 | Gibberellin insensitive dwarf1 | Similar to hormone-sensitive lipase |
| GLR1.1 | Glutamate receptor 1.1 | Glutamate-gated ion channel family |
| GRF1 | General regulatory factor 1 | 14-3-3 chi |
| GRF3 | General regulatory factor 3 | 14-3-3 psi |
| GRF8 | General regulatory factor 8 | 14-3-3 kappa |
| HB52 | Homeobox protein 52 | homeodomain-leucine zipper (HD-Zip) class I TF |
| HB54 | Homeobox protein 54 | homeodomain-leucine zipper (HD-Zip) class I TF |
| HKL1 | Hexokinase-Like1 | Hexokinase |
| HT1 | High leaf temperature 1 | Raf kinase |
| HXK1 /GIN2 | Hexose kinase/Glucose insensitive 2 | Hexokinase |
| HXK7 | Hexose kinase 7 | Hexokinase |
| HY5 | Elongated hypocotyl5 | bZIP TF |
| IPTs | Isopentenyl transferase-related genes | CK biosynthesis genes |
| KIN10 | Catalytic subunit of the SnRK1 complex | CAMK Ser/Thr protein kinase family |
| LBD16/37/38/39 | Lateral organ boundaries domain 16/37/38/39 | LBD TF |
| MAX2 | More axillary growth 2 | F-box protein |
| MPK4/MPK12 | Mitogen-activated protein kinases | MAP kinases |
| NAC089 | NAM/AF1/2/CUC2 | NAC TF |
| NLP7 | Nodule inception-like protein 7 | NIN-like protein |
| NPF5.12/TOB1 | Peptide transporter family (NPF)5.12/Transporter of IBA 1 | Major facilitator superfamily (MFS) |
| NRT1.1/NPF6.3 | Nitrate transporter (NRT) 1.1 /peptide transporter family (NPF) 6.3 | Major facilitator superfamily (MFS) |
| NRT2 | Nitrate transporter (NRT) 2 | Major facilitator superfamily (MFS) |
| NRT2.1/2.2/2.4/2.5 | Nitrate transporters (NRT) 2.1/2.2/2.4/2.5 | Nitrate transporters |
| O2 | Opaque 2 | bZIP TF |
| OsGRF4 | Growth-regulating factor 4 | TF |
| OsNGR5 | Nitrogen-mediated tiller growth response 5 | AP2 TF |
| PII | - | Homotrimeric signal transduction proteins |
| PAP1 | Production of anthocyanin pigment1 | MYB TF |
| PIF | phytochrome-interacting factor | bHLH TF |
| PIN2 | Pin-formed 2 | Auxin efflux facilitator |
| PP2C | Protein Phosphatase Type 2C | ABA signalling inhibitor |
| PPC2 | Phosphoenolpyruvate carboxylase 2 | Oxalacetate biosynthesis |
| PYL | Pyrabactin Resistance-Like | ABA receptor |
| PYR1 | Pyrabactin Resistance 1 | ABA receptor |
| RALF1 | Rapid alkalinization factor peptide | Secreted peptide |
| RCAR | Regulatory Component of ABA Receptor | ABA receptor |
| ROP2 | Rho-related protein 2 | Small GTPase |
| RPT5B | 26S proteasome AAA-ATPase subunit | Proteasome ATPase |
| S6K | RIBOSOMAL PROTEIN S6 KINASE | Ribosomal kinase |
| SLR1 | Slender rice 1 | GA signalling repressor |
| SnRK1 | Sucrose non-fermenting 1 (snf1)-related kinase 1 | Kinase complex |
| SnRK2 | SNF1-related protein kinase 2 | Serine/threonine kinases |
| SUGCAR1 | Sucrose and Glucose Carrier 1 | Major facilitator superfamily (MFS). NTR1/NPF-type |
| SUT/SUC | Sucrose transporters | sucrose/H+ symporters |
| SWEET | Sucrose will eventually be exported | Sugar transporter |
| TOR | Target of rapamycin | Kinase complex |
| TPS1 | Trehalose-6-phosphate synthase | T6P synthesis enzyme |
| VAR2 | Variegated 2 | FtsH metalloprotease |
| VHA-B1 | Vacuolar H+-ATPase B1 | H+-ATPase |
| WRI1 | Wrinkled1 | AP2/EREBP TF |
| WUS/WOX | Wuschel (WUS)/wuschel-related homeobox (WOX) | Homeodomain TF |
